# Oculometric Measurement of Concussion Magnitude in Professional Baseball Catchers

**DOI:** 10.3390/brainsci16040369

**Published:** 2026-03-29

**Authors:** Richard Baird, Ryan Harrison, Quinn Kennedy, Mollie McGuire, Dorion Liston

**Affiliations:** 1 United States Air Force Academy, Colorado Springs, CO 80840, USA; richardbaird20.20@gmail.com; 2 Slow The Game Down, Laguna Beach, CA 92651, USA; ryan@slowthegamedown.com; 3 neuroFit, Inc., Mountain View, CA 94040, USA; qkennedy@neurofit.tech; 4 Naval Postgraduate School, Monterey, CA 93943, USA; mrmcguir@nps.edu

**Keywords:** concussion, oculometrics, athletes, TBI, vision rehabilitation

## Abstract

**Background/Objectives**: Due to their positions, professional baseball catchers are at elevated risk of concussion, which can impair visual processing. There is a need for sensitive sensorimotor monitoring tools to track concussion-related neurophysiological changes more accurately. We investigated whether oculometrics can address this need. **Methods**: Four Major League Baseball catchers completed an oculometric assessment shortly after suffering a concussion (Time 1) and again after completing vision rehabilitation (Time 2). The assessment produces 10 *z*-scored measures, including a summary score. **Results**: Players’ Time 1 summary score tended to be typical of a normal healthy adult (Mean = 0.07 *z*-scored units). On average, players improved by 1.3 z-score units from their Time 1 summary score (SD = 1.07). Exploratory analyses revealed that sensorimotor recovery was driven by smooth pursuit latency, proportion of tracking comprising smooth pursuit, and the amplitude of catch-up saccades. **Conclusions**: Our analysis was based on a very small sample of concussion cases, each of which was unique. Despite this limitation, our data show how oculometrics can measure improvements in visual processing following a concussion among baseball players with exceptional perceptual-motor skills. Our data highlight the risk that brain injuries in high-performing individuals go undetected due to standard-of-care tools normed to behavior from healthy control populations; for these athletes, “normal” scores cannot be interpreted as neurologically “healthy”.

## 1. Introduction

Like many sports, excellent baseball performance requires best-in-class visual and motor skills, alone and in tight coordination [1,2]. Professional and college baseball players have superior fixation stability [3], convergence latency [4], vestibulo-ocular reflexes [5], visual acuity, contrast sensitivity, and visual tracking [6,7] compared to non-athletes, and professional players have better visual acuity and depth perception than collegiate players [8]. Over time and with increased expertise, positions show differential performance; at the collegiate level, hitters and pitchers show equivalent visual ability, but at the professional level, hitters show better depth perception than pitchers [8]. Baseball hitters must hone their visuomotor skills to compete at high levels of play, despite occupational risks.

For catchers, concussions are prevalent. There were 110 documented concussions during the 2010–2018 Major League Baseball (MLB) seasons, with catchers accounting for the largest share at 38% [9] at an estimated rate of three concussions per 100,000 pitches [10]. To put this statistic in context, each team throws an average of 146 pitches per game and plays 162 games per season, totaling 23,652 exposures during regular season games, excluding spring training, warmups, and practices. With 32 MLB teams, this predicts 23 concussions per season for MLB catchers. Across all positions, players average nine days of missed time due to any type of concussion, which more than doubles for injuries caused by the ball [10]. A total of 20% of all concussed players lost extensive playing time or ended their careers early [9].

Concussions can impact sensorimotor skills, such as attention, visual tracking, and proprioception [11,12], which professional athletes depend upon. One study compared perceptual motor performance between people who had a clinically-diagnosed concussion but were no longer experiencing concussion-related symptoms to a group of age- and sex-matched controls. Two subtle sensorimotor decrements emerged. First, the concussion group used compensatory strategies for simple perceptual motor tasks that led to equivalent performance as the controls. Second, when the task became more temporally demanding, the concussion group performed worse than the control group [11]. As the average time since the last concussion was 3.5 years, these results indicate prolonged neurological impact.

Critically, sensorimotor impairments left undetected and untreated can increase subsequent risk for head and neck injuries [13,14]. Even after athletes have met the “return to sport” criteria with a standard neurological exam, they can have subtle long-term neuroanatomical and neurophysiological impairments that affect perceptual motor efficiency and increase the risk for subsequent concussion and musculoskeletal injury [13,15]. These findings indicate that there is a need for more sensitive measures to detect signs of concussion, evaluate treatments, and monitor recovery. Precision measurement tools for subtle sensorimotor impairments are critical for high-performing cohorts at elevated risk due to sports or military occupational specialty.

Oculometric assessment can fill this gap. Eye movements provide objective, noninvasive, and automated biomarkers of attention, processing speed, and spatial perception [16]. Previous research found that oculometric impairment occurs in up to 90% of concussion cases [12]. Our work demonstrates that oculometrics can measure a wide range of sensorimotor functions and can detect the presence and severity of TBI [17,18]. Thus, oculometrics may be particularly helpful for objectively quantifying concussion-related signs in those with excellent sensorimotor skills, as well as monitoring improvement during rehabilitation. Our case series reports the use of a validated oculometric TBI assessment method to quantify post-concussion changes in four MLB catchers.

## 2. Materials and Methods

Four MLB catchers completed a five-minute visual tracking assessment shortly after suffering a concussion (Time 1) and again several weeks later (Time 2) after they participated in a vision rehabilitation program that collected this data. The analysis of this data was determined not to be human subjects research by the Naval Postgraduate School IRB (NPS.2024.0185-DD-N).

### 2.1. Vision Rehabilitation

Vision rehabilitation consisted of two parts. First, a comprehensive vision performance evaluation was conducted with an emphasis on skills directly tied to catcher performance. The evaluation protocol included measures of stereopsis, binocularity and fusion range, accommodative facility, suppression, convergence/eye teaming at varying distances, and saccadic eye movements in both primary gaze and alternating over-the-shoulder conditions. Instruments such as vectograms (Stereo Optical, Chicago, IL, USA), Random Dot E tests (Stereo Optical, Chicago, IL, USA), and Eye Trac (Slow the Game Down, Irvine, CA, USA) were employed to assess binocularity, suppression, and convergence ranges.

Second, individualized training interventions were developed, targeting the weakest skill areas identified during evaluation. The four injured catchers commonly demonstrated accommodative dysfunction, restricted binocularity, or limited eye-teaming ability. Intervention strategies included accommodative flipper exercises (Bernell, Mishawaka, IN, USA) to enhance focusing dynamics, depth perception cards (Slow the Game Down, Irvine, CA, USA) and vectograms (Stereo Optical, Chicago, IL, USA) to improve stereopsis and fusion. Visual performance training posters (Slow the Game Down, Irvine, CA, USA) and Eye Trac (Slow the Game Down, Irvine, CA, USA) activities were integrated to reinforce accuracy and efficiency of saccadic tracking under sport-specific conditions. Training progressed systematically, with emphasis placed on transferability to game-like demands and measurable improvements in visual comfort, efficiency, and performance.

### 2.2. Oculometric Assessment

We used a standardized, automated five-minute assessment of dynamic vision [19] using a step-ramp tracking task [20]. Each of the 45 trials was initiated when the subject fixated a central (0.5 deg diameter) black dot. Following a randomized interval (truncated exponential distribution, mean: 700 ms, range: 200–5000 ms), the dot made a small position step (displacement of 200 ms of target motion) in a random direction around the unit circle and began moving back toward the initial fixation location at a fixed speed drawn from a uniform distribution (16–24 deg/s). Subjects were verbally instructed to track the motion of the dot for as long as it was visible. Following completion of the 45 trials, the device ran an automated analysis to report a vector of 10 metrics including smooth pursuit latency, acceleration, gain, proportion smooth tracking, saccadic amplitude, direction noise, speed tuning responsiveness, and speed noise. A summary score called nFit was computed by transforming each measurement into a z-score referenced to a previously collected normative control sample [19], averaging the vector while accounting for the covariance. The normative control sample consisted of 41 cognitively healthy participants aged 20 to 56 years (median age: 27 years). Test–retest reliability was validated with a subset of participants who completed the oculometric test five times in one day. Within-person variability was between 3.5 and 25.1 times smaller than between-person variability. The formula for nFit yields a normal distribution centered at a mean of 0 and standard deviation of 1 for the normative control population.

### 2.3. Equipment

The oculometric assessment was run on neuroFit eye-tracking hardware (neuroFit, Mountain View, CA, USA), an oculometric device that can display the stimulus sequence for any standardized oculomotor protocol [3,19,21], collect high-quality monocular or binocular eye-position data, and compute summary metrics. The non-invasive eye-tracking system collects high-precision 2D eye-position signals (noise level of 0.2 deg in human control subjects, 0.01 deg for a glass eye) using two 850 nm IR illuminators and a central image sensor. Using an ergonomic chinrest (neuroFit, Mountain View, CA, USA), the subject’s head is stabilized at a view distance of 57 cm from the LED-backlit LCD display (24” display, 1920 horizontal, 1080 vertical, 120 Hz). Using an automated module for detection and registration of facial features, the system can track one or both eyes for any subject within the 5.35–7.25 cm anthropometric range of inter-pupillary distance. We used 250 Hz monocular tracking of the left eye in all participants to allow high sampling rates that would have been halved to 120 Hz using binocular tracking, and to allow continuity with previous methods [17,19].

### 2.4. Procedures

Within a few days of receiving a concussion diagnosis from an impact event during play, catchers were referred for oculometric assessment (Time 1). They then underwent a period of vision rehabilitation therapy (Slow the Game Down, Irvine, CA, USA) which varied by player, ranging from 15 to 75 days. The oculometric task was repeated at the end of rehabilitation (Time 2).

## 3. Results

### 3.1. General Trends

**Figure 1:**
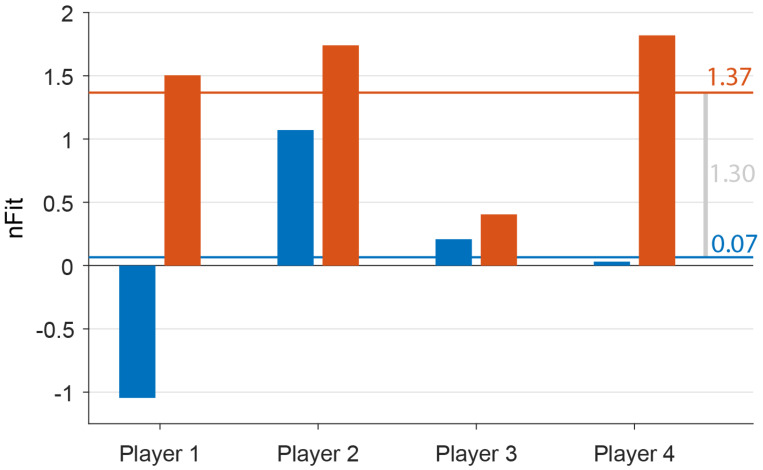
Oculometric performance before and after vision rehabilitation. Blue bars represent oculometric data collected following a recent concussion (Time 1); most players performed similarly to a typical healthy adult. The horizontal blue line represents the average nFit score of 0.07. Orange bars represent nFit scores after vision rehabilitation (Time 2); most players performed noticeably better than the typical healthy adult. The horizontal orange line represents the Time 2 average of 1.37, yielding an average improvement of 1.30.

As depicted in Figure 1, on average, players’ nFit score improved from 0.07 at Time 1 to 1.37 at Time 2. This overall nFit increase of 1.3 (SD = 1.07) was driven by improvements in all individual metrics, including latency (mean improvement: 9.75 ms, 95% confidence interval: −0.26 to 19.76), speed noise (mean improvement: 1.25 deg/s, 95% confidence interval: −0.62 to 3.12), gain (mean improvement: 0.08, 95% confidence interval: 0.02 to 0.15), and saccadic amplitude (mean improvement: 0.6, 95% confidence interval: −0.14 to 1.33).

**Figure 2:**
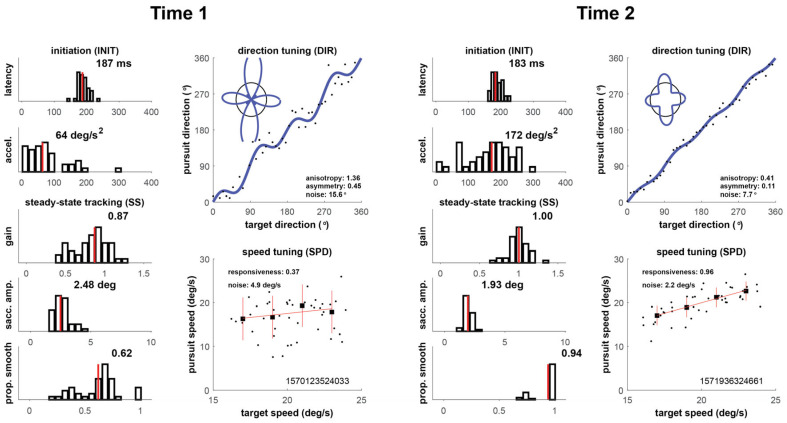
Oculometric changes for Player 1. The left-hand panel shows a summary figure at Time 1, shortly after suffering a concussion and prior to starting vision rehabilitation. The right-hand panel shows corresponding summary figure at Time 2.

**Figure 3:**
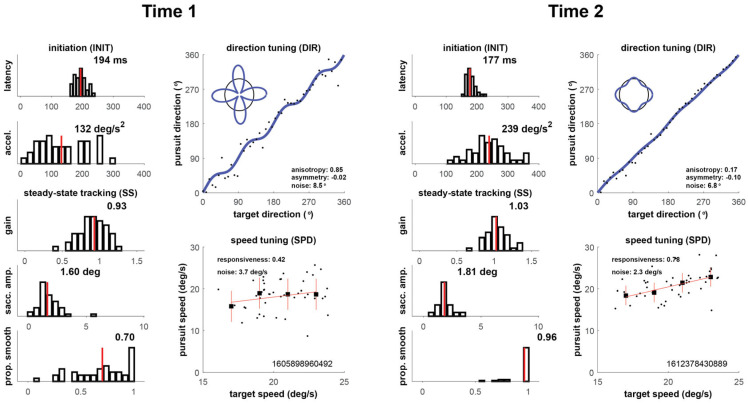
Oculometric changes for Player 2.

**Figure 4:**
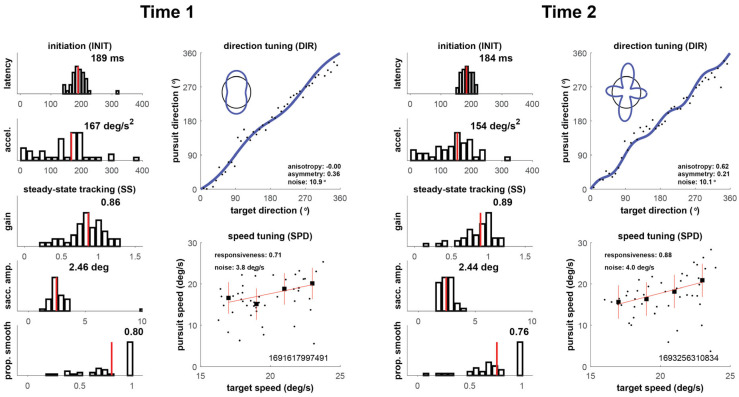
Oculometric changes for Player 3.

**Figure 5:**
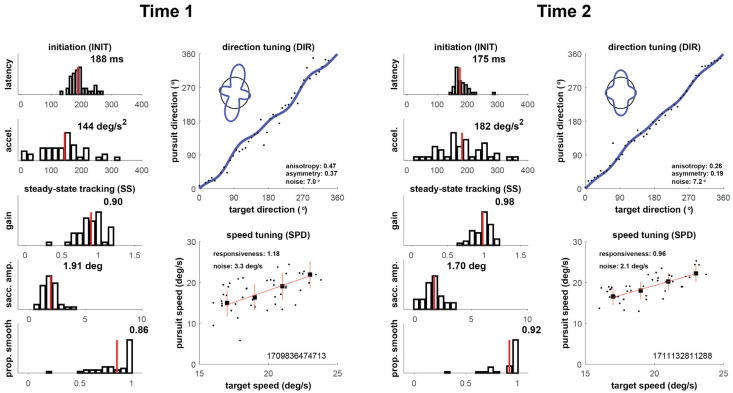
Oculometric changes for Player 4.

Our four cases are shown in Figure 2, Figure 3, Figure 4 and Figure 5 below, which depict summary figures from our visual tracking task at Time 1 and Time 2. The task requires the subject to release fixation and initiate tracking, initially driven by a negative feedback mechanism using a “retinal slip” error signal, yielding latency and acceleration metrics. Latency is the elapsed time from stimulus motion onset to the tracking response (top left graph), and acceleration quantifies the vigor of the ramp up in eye velocity (graph below latency). As the movement stabilizes during steady-state tracking, the negative feedback mechanism is no longer the primary driver of the movement, which transitions to a positive feedback mechanism that uses the percept of object motion encoded in cortical circuits. The metrics that characterize this epoch of the movement include gain, saccadic amplitude, and proportion smooth, in addition to direction noise, speed responsiveness, and speed noise. Gain is the ratio of eye velocity to target velocity (graph below acceleration), saccadic amplitude quantifies the size of saccadic interruptions of smooth pursuit (graph below gain), and proportion smooth gives the ratio of total eye displacement consisting of smooth pursuit (bottom left graph). Direction tuning (top right graph) and speed tuning (bottom right graph) quantify misperceptions in direction and speed, respectively. Complete descriptions of each of the metrics can be found in the Supplementary Materials.

#### 3.1.1. Player 1

As shown in Figure 2, Player 1 showed the most severe initial impairment as well as the largest improvement. Twenty-one days elapsed between Time 1 and 2. His Time 1 nFit score was 1 SD below the expected control subject (*z* = −1.04). Performance was particularly impaired on acceleration (*z* = −1.58) and direction noise (*z* = −2.08), with speed responsiveness, speed noise, and latency all impaired by about 1 SD. Gain (*z* = 0.65), saccade amplitude (*z* = −0.36), and proportion smooth were all relatively normal. At Time 2, his overall performance increased to an nFit score of 1.50, an improvement of more than 2.5 *z*-units, with individual Time 2 metrics ranging from −0.44 (latency) to 3.01 (proportion smooth). The level of internal noise in both direction and speed improves by a factor of 2 between the timepoints.

#### 3.1.2. Player 2

As shown in Figure 3, Player 2’s Time 1 nFit score of 1.07 was 1 SD above the normal population, with nearly all individual metrics showing preserved function or the ability to compensate for the concussion. However, latency (*z* = −1.17) was profoundly impacted, especially in the context of all other metrics, which ranged from z-scores of 0.1 (speed noise) to 0.5 (acceleration and direction noise) to 1.0 (gain) to above 2.0 (proportion smooth and speed responsiveness). His Time 2 oculometric performance improved to a nFit score of 1.74, with latency improving by almost 2 SD. All individual metrics improved to z-scores greater than 1.0, with the exception of latency (*z* = 0.73) and direction noise (*z* = 0.42). The elapsed time between Time 1 and 2 is 15 days.

#### 3.1.3. Player 3

As shown in Figure 4, oculometric performance showed a small improvement between Times 1 and 2 for Player 3. Nineteen days elapsed between Time 1 and 2. His Time 1 nFit score was 0.21; at Time 2, it increased to 0.40. At both time points, several individual metrics (acceleration, gain, speed responsiveness) fell far above the median of the control population; however, latency (*z* = −0.58) and direction noise (*z* = −0.43) remained impaired. This player demonstrated a persistent “mixed” injury profile with impairments in specific metrics (e.g., latency, direction-tuning noise, speed-tuning noise) and no loss of performance in other metrics.

#### 3.1.4. Player 4

As shown in Figure 5, Time 1 oculometric performance was consistent with that of a normal control individual, with an nFit score of 0.03 and most individual metrics falling within 0.5 SD of 0.0. There were 75 days between Time 1 and 2. Tellingly, latency showed strong impairment (*z* = −2.04) but was balanced by excellent gain (*z* = 1.38) and saccade amplitude (*z* = 1.27). At Time 2, performance improved by almost 2 full *z*-units with a nFit score of 1.79, and all metrics were above the expected control range. Improvements in individual metrics were most notable for latency, acceleration, asymmetry, and proportion smooth, with improvements of 2.0 SD or greater.

#### 3.1.5. Comparison of Concussions

The hallmark of the four concussion cases at Time 1 was prolonged latency, which fell below the expected control value in all cases. All other metrics were either spared or compensated for the injury in these high-performing athletes. In the aggregate score, nFit at Time 1 ranged from 1 z value below normal (Player 1) to 1 z value above normal (Player 2), which masked telltale variability in individual metrics. In terms of individual metrics, Player 1 performed well below a normal control population on approximately half of the metrics, whereas Players 2, 3 and 4 performed below 0.0 only on one or two metrics. Individual metrics at or above the normal control population also varied by player, from two metrics (Player 4) to eight metrics (Player 2). A consistent pattern was that all had above-average gain function, and three were also at or above the normal saccadic amplitude.

Improvements on nFit at Time 2 also ranged broadly from 0.2 (Player 3) to 2.5 (Player 1). Lower Time 1 nFit score did not necessarily lead to greater improvement: Player 2, with a Time 1 nFit score of 1.07, improved more than Player 3, whose Time 1 nFit score was 0.20. Finally, although most players showed improvement on almost all metrics, Player 3 still showed impairment on some metrics and very little change overall.

## 4. Discussion

In our case series report, four MLB catchers suffered from concussions with quantifiable visuomotor signs. On average, their immediate post-concussion oculometric performance was typical of a normal control participant. After receiving vision rehabilitation therapy and navigating the natural healing process, their oculometric performance tended to increase substantially. However, both initial scores and areas of improvement varied across players.

These results lead to four conclusions. First, injuries in high-performing populations can show a mixed pattern of individual oculometrics. Whereas latency, direction-tuning noise, and speed-tuning noise tended to show substantial impairment, acceleration, gain, saccadic amplitude, and proportion of smooth tracking can remain intact and compensate for impaired ocular functions.

Second, for high-performing individuals, achieving “normal” control scores does not equate to being neurologically healthy. These individuals may achieve an average-looking score while still being far below their own operating range. For example, all players showed exceptional performance (at least 2 SD above a normal control population) on individual metrics after vision rehabilitation, suggesting that their typical performance range is well above that of the typical person. Thus, high-performing populations are at elevated risk of undetected TBI when using tools that only detect impairment below the level of normal control populations, especially without a baseline. To mitigate this risk, individual baselines taken at a frequency appropriate to the exposure (e.g., yearly, monthly, or weekly) improve the statistical power to detect injury.

Third, the detailed vector of oculometrics may be a useful method to assist in the diagnosis, treatment and monitoring of concussion and, particularly for high performing individuals, can reveal disrupted motion-processing circuitry. It can aid vision rehabilitation by pinpointing specific visuomotor functions that have been compromised.

Fourth, vision rehabilitation may contribute to recovery from subtle neurological impairments, which in turn reduces the risk of follow-on injury [22]. When a baseline assessment is available, oculometric change scores such as those shown in Figure 1 can be used both to gauge both the magnitude of brain injury and also the magnitude of the associated recovery, which are conflated in our current data

We note several limitations with this dataset. Prior concussion history, concussion severity, baseline oculometric performance (i.e., pre-injury data), and protective equipment worn at the time of the concussion were not collected, limiting our understanding of the injuries and vision rehabilitation effectiveness. We should point out that these conditions of incomplete information are most often the case when concussion and brain injury cases present in clinics and training facilities, emphasizing the need for additional characterization of oculometric signs of brain injury in much larger samples. A prospective cohort design would be needed to capture pre-injury baselines in samples with elevated risk, like artillery soldiers or professional baseball catchers. Following these samples forward in time during risk exposure allows discrete injuries to be characterized and quantified more precisely than we were able to, and any cumulative effects to be tracked longitudinally. Additionally, due to the nature of injury cases and the varied impact on visuomotor function across the four catchers, it is unclear to what extent oculometric improvements can be attributed to the rehabilitation intervention versus natural recovery processes. A randomized trial including a control group would be needed to evaluate the therapeutic effects of vision rehabilitation, or any other intervention, over and above natural recovery in concussed patients.

## 5. Conclusions

Our analysis is based on a very small sample of concussion cases and each injury was unique. Despite this limitation, our data show how oculometrics can track and quantify improvements in sensorimotor processing following a concussion among individuals with exceptional sensorimotor abilities. These data provide context for the oculometric presentation of brain injury in high-performing populations; variability in z-scores for individual metrics can be more informative than the composite summary score. Consistent with previous findings [13,15], these data suggest that high-performing populations are at elevated risk of subtle sensorimotor impairment due to TBI.

## Data Availability

The data presented in this study are available on request from the corresponding author due to privacy restrictions related to subject identifiability.

## Supplementary Materials

The following supporting information can be downloaded at: https://www.mdpi.com/article/10.3390/brainsci16040369/s1. Supplemental Table S1. Operational definitions of individual oculometrics for two intervals of tracking.

## Abbreviations

The following abbreviations are used in this manuscript:

MLB	Major League Baseball
TBI	Traumatic Brain Injury
SD	Standard deviation
