# Supplementary material for: Oculometric Measurement of Concussion Magnitude in Professional Baseball Catchers
Source: Brain Sci. 2026 Mar 29;16(4):369. doi: 10.3390/brainsci16040369 (PMC13114181; doi:10.3390/brainsci16040369)
Supplement: Supplementary file 1 [file brainsci-16-00369-s001.zip › Supplemental Table.pdf]

Metric	Operational Definition
Latency	The elapsed time from stimulus motion onset to the tracking response, when eye velocity departs from zero.
Acceleration	When the eye starts to move, acceleration quantifies the vigor of the ramp up in velocity.
Gain	The ratio of eye velocity to target velocity.
Saccadic Amplitude	The median size of saccadic interruptions during smooth pursuit.
Proportion Smooth	The proportion of total eye displacement consisting of smooth pursuit; the remaining displacement consists of saccadic interruptions.
Direction Noise	The level of internal noise in direction perception, quantifying the standard deviation of misperceptions on a trial-by-trial basis.
Speed Responsiveness	The sensitivity to trial-by-trial differences in target speed. A responsiveness of zero means that all target speeds look approximately the same.
Speed Tuning	The level of internal noise in speed perception, quantifying the standard deviation of misperceptions on a trial-by-trial basis.

Supplemental Table S1. Operational definitions of individual oculometrics for two intervals of tracking. **Initiation metrics** (latency and acceleration) quantify the early phase of the tracking movement. These are measured by fitting a two-parameter hinge model to eye velocity, when fixation is released and movement is driven by a negative feedback "retinal slip" error signal, the difference between eye velocity and target velocity. **Steady-state metrics** (gain, saccadic amplitude, proportion smooth, direction noise, speed responsiveness, speed tuning) quantify the later phase of the tracking movement, measured from the eye position traces during a fixed interval from 400 to 700 ms following motion onset. The later period of tracking is sustained by the percept of object motion, a positive feedback loop that incorporates cortical, brainstem and cerebellar circuits.
